# Saltiness and Bitterness Perception of KCl-Based Mixed Salts (KCl/NaCl/L-Arginine) Evaluated by the R-Index and Partial Projective Mapping Methods

**DOI:** 10.3390/foods15091605

**Published:** 2026-05-06

**Authors:** Busarawan Chaiya, Pitchayapat Chonpracha, Pamarin Waimaleongora-ek, Sujinda Sriwattana, Witoon Prinyawiwatkul

**Affiliations:** 1 Department of Food Technology and Nutrition, Faculty of Natural Resources and Agro-Industry, Kasetsart University, Chalermphrakiat Sakhon Nakhon Province Campus, Sakon Nakhon 47000, Thailand; busarawan.ch@ku.th; 2 School of Nutrition and Food Sciences, Louisiana State University, Agricultural Center, Baton Rouge, LA 70803, USA; pitchayapat_chonpracha@hotmail.com (P.C.); pamarin@gmail.com (P.W.-e.); 3 Product Development Technology Division, Faculty of Agro-Industry, Chiang Mai University, Chiang Mai 50100, Thailand; sujinda.s@cmu.ac.th

**Keywords:** salt substitute, sodium reduction, potassium chloride, R-Index, partial projective mapping

## Abstract

KCl, when used at a high concentration, imparts undesirable bitterness which can be suppressed using blockers such as L-Arginine (L-Arg). Sensory discrimination of saltiness and bitterness intensity of salt mixtures (KCl/NaCl/L-Arg) was determined by two methods: the R-Index (RIX) and Partial Projective Mapping (PPM). Four mixed salt solutions [KCl/NaCl/L-Arg: (A) 70/20/10, (B) 65/25/10, (C) 60/30/10, and (D) 55/35/10] at 0.5%, 1.0% and 1.5% *w/v* were prepared in order to impart different saltiness and bitterness perception, and compared to the control [100% NaCl; 0:100:0 (E)]. Using RIX, some degrees of differences in perceived saltiness and bitterness among salt mixtures were detected at all salt concentrations. However, using PPM, significant differences in saltiness and bitterness perception were not found between all salt mixtures and the control at 0.5% *w*/*v*. Both RIX and PPM methods could be used to discriminate saltiness and bitterness intensity among salt substitute mixtures and the control; however, the obtained results were conceptually distinct and must be interpreted cautiously based on the experimental test conditions used in this study.

## 1. Introduction

A high-sodium diet has been associated with high blood pressure, cardiovascular disease and kidney failure, whereas reducing sodium intake can help reduce hypertension and improve health consequences [1]. Widespread consumer concern regarding salt intake has made it an important goal for the food industry to reduce the sodium content in food products [2]. Reformulation of food products has been identified as one of the most cost-effective strategies to lower dietary sodium intake at the population level [3]. However, successful implementation of sodium reduction programs may not concur with the consumer’s innate preference for salty tasting food, and, hence, causes negative changes in consumer perception [4].

Several techniques have been applied with regard to sodium reduction [2,3,5,6,7,8,9,10,11,12,13,14]. Partial replacement of NaCl with KCl is the most common method [15] due to their similarities in molecular composition. KCl imparts a salty taste to the product which differs from that of NaCl [16]. The maximum level of KCl as a salt replacer is also an important consideration because levels above 30% can impart a bitter flavor, along with a metallic aftertaste [17,18], while 40% sodium replacement may result in an unacceptable taste [19,20].

One method that has been frequently studied is the use of a bitterness blocker to interfere with or inhibit bitter taste receptor activation [21]. Some low molecular weight compounds, including amino acid derivatives and peptides, have been reported to mask bitter tastes [9,22]. However, it is not known if the bitter taste masking mechanisms take place at the receptor level or on the intracellular components of the taste signaling cascade [23]. Ogawa et al. (2004) [24] reported that L-Arginine could reduce bitterness at a low concentration (0.003%) of a quinine solution, and this effect was more pronounced in the presence of NaCl. The mechanism of the L-Arg masking effect was reported in relation to its guanidinium functional group and the sodium channel in the human taste bud. Therefore, studies related to the bitterness-suppressing effects of L-Arg on sodium replacement with KCl at varying salt substitute concentrations are promising.

Sensory discrimination methods should be carefully selected, depending largely on the specific objectives, the complexity of test products, and the sample size (i.e., the number of panelists) [25]. The RIX method is a non-parametric analysis method used to measure the degree of difference (%) between two samples [26]. It can be calculated from rank data when ranking among products is practical, that is, the test samples can be ranked based on a specific attribute and a given dimension. Some advantages of RIX include the computational simplicity of data analysis and its flexibility for use in a wide variety of test protocols [27]. Projective mapping (PM) is a rapid profiling method that has been applied to a wide variety of applications and can produce an overview of many products [28]. Furthermore, this method has been used with participants who have a varied knowledge of sensory evaluation or the samples being tested. Pfeiffer and Gilbert (2008) [29] modified the original version of PM and named it Partial Projective Mapping (PPM). For PPM, the panelists evaluate similarities or differences in products in a specific modality (such as appearance, flavor, texture), as opposed to global similarities or differences. PPM was reported to demonstrate better discrimination than PM and showed a higher correlation with conventional descriptive analysis [30].

Crouch et al. [31] evaluated the taste acceptability of several KCl-enriched salt substitute blends (35%KCl/65%NaCl, 50%KCl/50%NaCl, 66%KCl/34%NaCl, and 100% KCl) placed on crackers without a bitterness blocker. They reported that the taste of the 50% KCl salt substitute would be well tolerated. In the current study, the main objective was to use the RIX method to evaluate the saltiness and bitterness perception of mixed salt (KCl/NaCl/L-Arg) solutions with a high level of KCl (55%, 60%, 65% and 70% *w*/*v*) in the presence of L-Arg, a bitterness blocker. With the existing knowledge of PPM, it may be suited for simple and quick sensory discrimination among mixed salts. Hence, the second objective of this study was to test a hypothesis that PPM was able to detect differences in saltiness and bitterness among mixed salt (KCl/NaCl/L-Arg) and NaCl solutions, and to provide results comparable to those from RIX.

## 2. Materials and Methods

### 2.1. Preparation of Mixed Salt Solutions

The FCC-grade NaCl and L-Arg were purchased from Voigt Global Distribution, LLC (Kansas City, MO, USA), while the FCC-grade KCl was obtained from EMD Chemicals, INC. (Gibbstown, NJ, USA). The Brita Water Filtration System (Brita Products Company, Oakland, CA, USA) was purchased from a local supermarket. Four mixed salt solutions (KCl/NaCl/L-Arg) and the control NaCl solution at 0.5%, 1.0% and 1.5% *w/v* were prepared as shown in Table 1. This salt concentration range was selected because many prepared or cooked products contain sodium levels that correspond to 0.5–1.5% NaCl [32]. A fixed 10% L-Arginine was within the range evaluated in our previous experiment, in which an ideal intensity of salt solutions containing NaCl (0–100%), KCl (0% to 100%), and L-Arginine (0% to 15%) was evaluated by consumers [33]. The water used for preparation of the salt solution was filtered to minimize undesirable taste or odor. Each mixed salt solution was poured into 2 oz plastic cups with lids labeled with three-digit numbers and stored at room temperature for further use. All samples were prepared one day before the sensory testing session.

### 2.2. RIX Analysis with Ranked Data

A panel of 20 untrained panelists (13 females and seven males) were recruited. They consisted of students, staff, and faculty from Louisiana State University (LSU), Baton Rouge, LA, USA. The sample size (n = 20 panelists × six independent replications) used in this study was adequate for estimations of RIX [27]. All samples were kept and served at room temperature (ca. 25 °C). A set of five 25 mL samples at the same concentration (0.5%, 1.0%, or 1.5% *w*/*v*) was presented in a counterbalanced design so that all samples appeared equally in each position, hence minimizing order and carryover effects. Each panelist was instructed to taste the sample in the order presented by swirling it in the mouth and expectorating it into the cups provided. After tasting all samples, they ranked the samples in order of taste intensity (1 = most intense; 5 = least intense), with no ties allowed. They then cleansed their palate with drinking water after tasting each sample. They were asked to take a bite from an unsalted wheat cracker and rinse their palate with water between samples to minimize carryover effects that could possibly be accumulated during the sessions. In each session, saltiness and bitterness of the mixed salt solutions were evaluated independently. After completing the first set, the samples were removed, and the panelist was required to take a 5 min break prior to evaluating the other taste modality. Eighteen sessions (three concentrations × six replications) were carried out on six different days. The sensory analyses were conducted in partitioned booths under white light in the Sensory Services Laboratory, School of Nutrition and Food Sciences, at LSU.

### 2.3. Partial Projective Mapping (PPM) Evaluation

The PPM analyses were conducted in a conference room in the Sensory Services Laboratory, School of Nutrition and Food Sciences, at LSU. Another group of thirty untrained panelists were recruited from LSU. In each session, all fifteen samples (three concentrations × five mixed salt solutions) were presented simultaneously to panelists with no specific presentation order. All samples were kept and served at room temperature (ca. 25 °C). Each panelist was provided with a white sheet of paper (60 cm × 60 cm). Panelists were given specific directions on how to perform the test before starting their own evaluation. They were informed that the two-dimensional sheet was constructed with a line scale range from no to strong intensity (0–100), where the horizontal and vertical axes represented saltiness and bitterness perception, respectively. Panelists were asked to evaluate all fifteen samples first and then placed the samples on the paper based on the sample’s similarities/differences they perceived in bitterness and saltiness. The samples considered similar in intensity would be placed closer and vice versa [34]. Panelists were asked to take a bite from an unsalted wheat cracker and rinse their palate with water between samples, and were instructed that re-tasting was allowed. Panelists performed this task at their own pace, however, within 40–60 min.

### 2.4. Statistical Analysis

RIX was computed to determine the effect of L-Arg on saltiness and bitterness perception of KCl-based mixed salt solutions at 0.5%, 1%, and 1.5% *w/v* concentrations. Rank data from six replications of each panelist was pooled and tabulated as frequency counts (see Table 2). The individual RIX was then calculated [27]. As an example, the below equation was used to calculate the RIX for a pair of sample A and E (the control):

$${RIX}_{A}=\frac{\left[a(g+h+i+j)+b(h+i+j)+c(i+j)+dj]+[\frac{1}{2}(af+bg+ch+di+ej)\right]}{\left(n_{A})(n_{E}\right)}.$$

The group RIX value was obtained by averaging the individual RIX values. A one-tailed test at a significance level of 0.05 was performed to determine the effect of L-Arginine on saltiness and bitterness perception in KCl-based salt solutions. The significance of RIX values was separately determined for saltiness and bitterness perception using the Tables reported by Bi and O’Mahony (2007; 2020) [35,36]. The mean rank data were calculated based on the rank data used to calculate the RIX values. Different superscript letters were assigned according to statistically significant differences among samples (*p* < 0.05) based on the critical RIX values.

For PPM, the numerical values (0–100; no–strong intensity) of the coordinates of x (saltiness) and y (bitterness) recorded on the sheet for each treatment from each panelist was measured considering the left bottom corner as the origin (0, 0). These numerical values were treated as continuous intensity ratings. Therefore, ANOVA was performed on these numerical values for each salt solution (x,y coordinate), and the differences between treatments were analyzed using Tukey’s HSD test at the α level of 0.05. Statistical analyses of PPM data were performed using statistical analysis software (SAS^®^ version 9.4, 2003). The number of significant pairs from RIX and PPM methods was then determined and compared.

## 3. Results and Discussion

### 3.1. Perception of Saltiness and Bitterness Using RIX

In this study, NaCl was used as the control or a ‘noise, *N*’ sample, which is known and expected to be saltier but less bitter compared to KCl, which was a ‘signal, *S*’ sample. For RIX, the assumption was that the ‘*S*’ sample was weak but stronger than the ‘*N*’ (i.e., *S* > *N*) [37], hence a one-tailed test was used in this study. The critical value of RIX indicating significant difference between samples at α = 0.05 was 64.4% (50 + 14.4%) for a one–tailed test [35]. The critical values for saltiness and bitterness were 35.6% (when *S* < *N*) and 64.4% (when *S* > *N*), respectively. Therefore, any calculated RIX values that were less than or equal to 35.6% for saltiness perception (Table 3) or greater than or equal to 64.4% for bitterness perception (Table 4) were statistically significant. To simplify the results from Table 3 and Table 4, the mean rank values with the designated significance of RIX of the salt solutions at 0.5%, 1% and 1.5% *w/v* concentrations are shown in Table 5.

Regardless of salt concentration, the KCl-based salt solutions were perceived as less salty and more bitter than the NaCl solution (Table 5). This finding was anticipated and supported by the relative salty taste index of KCl to NaCl of 0.6 by Guyton and Hall (2006) [38]. At the 0.5% *w/v* concentration, the salt solutions containing 55% KCl, 35% NaCl, and 10% L-Arg (sample D) were found to be different from the salt solution containing 70% KCl, 20% NaCl, and 10% L-Arg (sample A) in terms of saltiness (i.e., saltier). Besides the control, the salt solution containing the highest amount of NaCl (sample D) was perceived as saltier than other mixed salt solutions at the 1% *w/v* concentration. Moreover, the panelists were able to discriminate saltiness intensity when the difference in the ratio of NaCl in the solutions was ≥10% (for example, 35% vs. 25%) at the concentration of 1.0% and 1.5% *w/v* (Table 5). Thus, these findings confirmed that the presence of NaCl played a significant role in imparting a salty taste in the solutions with a sodium substitution level of more than 50% with KCl. Moreover, the higher the salt concentration, the more distinct the saltiness difference between the mixed salt solutions (Table 5).

Considering the bitterness perception, sample E, which was KCl-free, was perceived as the lowest in terms of bitterness intensity at all concentrations (Table 5). At the concentration of 0.5% *w*/*v*, panelists could not discriminate the bitter taste intensity among the KCl-containing solutions (*p* > 0.05). At the 1.0% *w/v* concentration, the salt solutions containing 55% to 60% KCl (samples C and D) were not significantly different in terms of bitterness, and neither were the salt solutions containing 65% and 70% KCl (samples A and B). However, the former pair (samples C and D) were less bitter than the latter pair (samples A and B), implying that panelists were able to differentiate the bitterness when the solutions contained more than 60% KCl at 1.0% *w/v* concentration. Moreover, when the salt concentration was increased from 0.5% *w/v* to 1.0% *w/v* and above, differences in bitterness intensity were observed in the sample pairs with a difference in KCl ≥ 10% (for example, 55% vs. 65%) (Table 5).

This finding is consistent with previous studies reporting that an increase in the level of NaCl replacement with KCl beyond 30–40% would result in an increase in bitterness and a decrease in saltiness [39,40]. Furthermore, Feltrin et al. (2015) [40] performed a temporal dominance of sensations to assess the dynamic sensory profile of aqueous solutions of NaCl and different sodium replacers. They reported that the saltiness of 1% KCl solution had a potency of 0.75 in relation to 0.75% NaCl solution, and the differences between NaCl and KCl for bitterness were discerned. With respect to our results, the panelists could differentiate the bitterness between mixed KCl-based salt solutions (all containing 10% L-Arg) and the control (KCl-free), implying that the concentration of L-Arg may need to be increased to obtain insignificant differences in bitterness. However, when considering KCl-based salt solutions at 0.5% *w*/*v*, the bitterness perception tentatively increased with increasing KCl, but the observed differences among samples were not significant (*p* > 0.05), which may partially be due to the presence of L-Arg. However, it cannot be ruled out that the absence of significant differences between some mixtures was due not only to a possible masking effect of L-Arg and the mixed salt concentration, but also to a limited sensitivity of the panel or the method under this current study’s conditions. At higher mixed salt concentrations, that is, 1.0% and 1.5% *w*/*v*, L-Arg was less effective in masking bitterness at a higher concentration of KCl (65–70%) in the salt mixture. Therefore, the masking effect of L-Arg depends on the mixed salt substitute concentration and the ratio of L-Arg in the salt mixture.

### 3.2. Perception of Saltiness and Bitterness Using PPM

Table 6 shows the intensity score of saltiness and bitterness of salt solutions at 0.5%, 1.0% and 1.5% *w/v* obtained by PPM. sample E was perceived to be saltiest among the salt solutions at 1.0% *w*/*v*. At 0.5% *w*/*v*, panelists perceived a similar saltiness intensity from all mixed salt solutions (*p* > 0.05), which was not significantly different from that of the control. This finding suggested that replacing NaCl with KCl at 55% to 70% (samples A–D) had no significant impact on the saltiness perception at 0.5% *w*/*v*. In contrast, the saltiness intensity increased with increasing salt concentration from 0.5% to 1.0% and 1.5% *w/v* (*p* < 0.05). At 1% *w*/*v*, the replacement of NaCl with KCl from 55% to 70% resulted in a noticeably lower saltiness perception compared to the control. At 1.5% *w*/*v*, samples (C and D) containing 55–60% KCl were saltier than the one containing 70% KCl (sample A); additionally, NaCl replacement with KCl up to 60% may avoid the noticeable difference in saltiness from the control.

Unlike saltiness perception, bitterness intensity was significantly affected by increasing KCl concentration in the salt substitute mixture (*p* < 0.05) but not by increasing salt concentration. At 0.5% *w*/*v*, there were no significant differences in bitterness perception among all salt solutions (*p* > 0.05) despite the tentatively increasing bitterness with increasing KCl concentration. One possible explanation was that either L-Arg successfully masked the bitter taste of KCl in the salt substitutes or the discrepancy between the bitterness intensities of the solutions was too minute to trigger differences to be perceived by panelists. As mentioned above, this absence of significant differences could possibly also be due to a limited sensitivity of the panel or the method under this study’s conditions. At 1.5% *w*/*v*, panelists could discriminate the bitterness between the salt substitutes and the control but could not differentiate the bitterness among the salt substitutes (Table 6).

**Figure 1:**
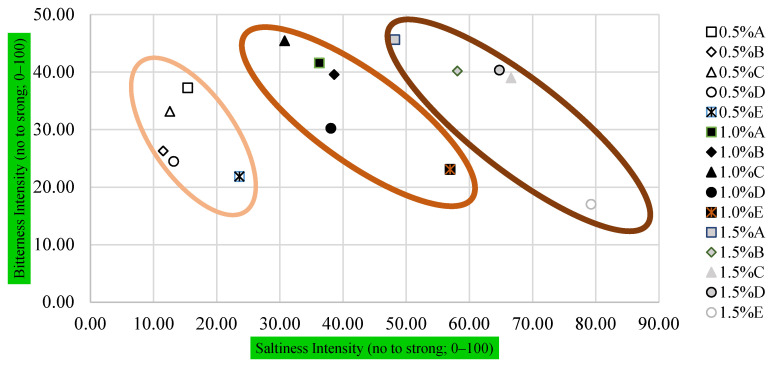
Graphical mapping of the saltiness and bitterness intensity of four KCl-based salt solutions and a NaCl solution grouped by concentration of 0.5% *w/v* (left), 1.0% *w/v* (middle), and 1.5% *w/v* (right) obtained from PPM. See Table 1 for description of samples A–E.

The results obtained from PPM can be simply visualized using graphical mapping (Figure 1) that depicts the sample’s position based on the panelists’ perception of bitterness and saltiness. Obviously, the mixed salt solutions could be classified into three groups following their concentrations. The KCl-based mixed solutions with 1.5% *w*/*v* were projected to the top far right of the x,y space (the higher bitterness and saltiness area), whereas the other two lower concentrations (0.5% and 1.0% *w*/*v*) were located far apart toward the left side. The control salt solutions at 0.5% *w*/*v*, 1.0% *w/v* and 1.5% *w/v* were positioned at the bottom–right of each concentration, indicating relatively lower bitterness and the highest saltiness intensity.

### 3.3. Comparison of Practicality and the Results of RIX and PPM

Practicality of RIX and PPM was compared in terms of the number of samples that could be tested and the time required to complete the task. PPM was performed with 15 samples evaluated all at once, hence at some points it may have led to carryover and sensory fatigue. In contrast, with RIX, five samples were served to panelists, so less fatigue may have been sustained. PPM was completed in approximately 40 min for all evaluations, whereas RIX took four times longer to complete all 15 samples within six sessions. The data analysis is included for practical comparison. For PPM, the mean intensity scores derived from the position (*x, y* coordinate) on the two-dimensional sensory space of saltiness and bitterness were computed and analyzed using ANOVA. On the other hand, the RIX values were derived from the rank data of the attribute intensity. The PPM data can be analyzed by parametric statistics such as ANOVA or multiple factor analysis (MFA) if various attributes are measured. Running data analysis with ANOVA or MFA can be quick and simple. PPM also provides a visual graphical mapping for product position, which may provide specific product profiles for many samples within a short time. However, one weakness of PPM when using a paper ballot is the time spent on data collection [28]. Measuring the product coordinates on the sheet of each panelist is tedious and time-consuming, particularly with a large sample size [41]. Like PPM, the ranking test with RIX analysis was also simple and fast by using the non-parametric statistics for the data analysis. The known advantage of the RIX method is that it can be used both as a good measure of effect size and as a powerful test statistic for sensory difference [36]. Theoretically, considering a relationship between RIX, the Mann–Whitney U test and the Wilcoxon Rank Sums Statistic, the RIX is only slightly less powerful than the *t*-test (about 5%), if the underlying distributions are normal [42]. When normal distribution assumptions do not hold, as would be expected with hedonic ratings [43,44,45], the RIX is frequently more powerful than the *t*-test. The measurement of RIX was unaffected by the decision criteria and the number of categories of rank data [27,37,42].

Although the two methods provided relatively similar findings in terms of saltiness and bitterness perception (Table 3, Table 4, Table 5 and Table 6), the RIX technique yielded more pairs with significant difference compared to PPM. In addition, the number of significant pairs between the RIX and PPM methods was slightly more for saltiness than bitterness (20 vs. 7 and 19 vs. 6, respectively) (Table 7). However, it would be premature to make conclusions regarding the superiority of RIX over PPM, and interpretation should be made cautiously. The two methods used were not applied under strictly comparable experimental conditions. Differences in the number of panelists, test structure, sample load, and session duration may have significantly influenced the observed discriminative capacity and sensitivity of the panelists. In particular, the PPM test with 15 samples in a single 40–60 min session may have caused sensory fatigue and carryover effects, particularly toward the perception of bitterness. Collectively, both RIX and PPM could be used to discriminate saltiness and bitterness intensity among KCl-based salt mixtures and the control; however, one must realize the distinct perceptual constructs captured by each method, that is, detectability versus perceptual relevance.

## 4. Conclusions, Research Gaps and Future Studies

A 50% sodium reduction level or higher can be challenging for food formulations. Based on the PPM results obtained, to yield the same perceived saltiness of NaCl, a higher concentration of salt substitutes containing KCl may be required. However, suppressing the bitterness of KCl using L-Arg was not significantly effective at a high level of KCl, as observed in a model solution evaluated in this study. Salt substitutes and bitterness blockers behave differently in various foods and beverages such as soups, breads, dairy products, snacks, and processed meats. Therefore, future studies should be performed in actual food and beverage matrices to determine whether the findings observed in this study hold true. Besides the sensory differences, consumer preference and acceptance of these foods containing KCl and L-Arg should also be evaluated. It would be interesting to investigate whether a higher level of L-Arginine in the salt substitute mixture might be sufficiently effective in masking the bitterness of KCl when used at higher levels.

This study revealed that both RIX and PPM could be used to evaluate the sensory perception of saltiness and bitterness of KCl-based mixed salt substitutes compared with the control NaCl solution. Both methods yielded complementary, but conceptually distinct, insights into saltiness and bitterness perception of the salt substitute. The RIX method primarily reflected the detectability of sensory differences while PPM offered overall perceptual relevance to these differences. Direct comparisons between RIX and PPM are rare in the literature. If the sensitivity of these two methods is to be compared, experimental conditions should be similarly set. In this study, only saltiness and bitterness perception were evaluated. Future studies should focus on how RIX vs. PPM perform across other sensory modalities, such as metallic and umami aftertastes. In addition, whether the performance of RIX vs. PPM differs across actual food matrices containing salt substitutes should be evaluated.

## Figures and Tables

**Table 1:** The ratio of KCl/NaCl/L-Arginine in the mixed salt solutions.

Sample	% KCl	% NaCl	% L-Arginine
A	70	20	10
B	65	25	10
C	60	30	10
D	55	35	10
E (control)	0	100	0

**Table 2:** Tabulated frequencies of rank data for individual RIX values.

Sample	Individual Response					Total
	1st (Most Intense)	2nd	3rd	4th	5th (Least Intense)	
A	*a*	*b*	*c*	*d*	*e*	*n_A_*
…	…	…	…	…	…	*…*
D	…	…	…	…	…	*n_D_*
E	*f*	*g*	*h*	*i*	*j*	*n_E_*

samples A–E indicate mixed salt solutions. See Table 1 for sample description. *a–j* indicate the cumulative frequency of rank intensity from six replications. *n* indicates total response of each sample.

**Table 3:** RIX values ^†^ of saltiness perception of different mixed salt solutions at varying concentrations.

Sample (KCl:NaCl:L-Arg)	Concentration (*w*/*v*)	Sample			
		B (65:25:10)	C (60:30:10)	D (55:35:10)	E (0:100:0)
A (70:20:10)	0.5%	0.424	0.384	0.350 *	0.024 *
	1.0%	0.409	0.327 *	0.226 *	0.029 *
	1.5%	0.417	0.302 *	0.217 *	0.051 *
B (65:25:10)	0.5%		0.471	0.439	0.031 *
	1.0%		0.379	0.258 *	0.038 *
	1.5%		0.378	0.279 *	0.043 *
C (60:30:10)	0.5%			0.454	0.042 *
	1.0%			0.319 *	0.046 *
	1.5%			0.372	0.067 *
D (55:35:10)	0.5%				0.053 *
	1.0%				0.079 *
	1.5%				0.065 *

^†^ Calculated based on 20 panelists × six independent replications. * Indicates RIX values (≤0.356) are significantly different (*p* < 0.05).

**Table 4:** RIX values ^†^ of bitterness perception of different mixed salt solutions at varying concentrations.

Sample (KCl:NaCl:L-Arg)	Concentration (*w*/*v*)	Sample			
		B (65:25:10)	C (60:30:10)	D (55:35:10)	E (0:100:0)
A (70:20:10)	0.5%	0.563	0.609	0.640	0.965 *
	1.0%	0.524	0.686 *	0.742 *	0.938 *
	1.5%	0.505	0.669 *	0.697 *	0.939 *
B (65:25:10)	0.5%		0.483	0.544	0.928 *
	1.0%		0.658 *	0.726 *	0.940 *
	1.5%		0.641	0.669 *	0.938 *
C (60:30:10)	0.5%			0.542	0.895 *
	1.0%			0.603	0.917 *
	1.5%			0.536	0.902 *
D (55:35:10)	0.5%				0.895 *
	1.0%				0.921 *
	1.5%				0.896 *

^†^ Calculated based on 20 panelists × six independent replications. * Indicates RIX values (≥0.644) are significantly different (*p* < 0.05).

**Table 5:** Mean rank scores of saltiness and bitterness perceptions of the mixed salt solutions at different concentrations.

Taste	Sample	KCl:NaCl:L-Arg	Mean Rank ^†^		
			0.5% *w*/*v*	1.0% *w*/*v*	1.5% *w*/*v*
Saltiness	A	70:20:10	3.82 ^c^	4.01 ^d^	4.01 ^d^
	B	65:25:10	3.48 ^bc^	3.73 ^cd^	3.72 ^cd^
	C	60:30:10	3.36 ^bc^	3.34 ^c^	3.24 ^bc^
	D	55:35:10	3.19 ^b^	2.73 ^b^	2.81 ^b^
	E	0:100:0	1.15 ^a^	1.19 ^a^	1.23 ^a^
Bitterness	A	70:20:10	2.19 ^a^	2.02 ^a^	2.23 ^a^
	B	65:25:10	2.57 ^a^	2.22 ^a^	2.32 ^ab^
	C	60:30:10	2.69 ^a^	2.86 ^b^	2.73 ^bc^
	D	55:35:10	2.93 ^a^	3.19 ^b^	3.05 ^c^
	E	0:100:0	4.63 ^b^	4.72 ^c^	4.68 ^d^

^†^ Mean rank scores (1 = most intense, 5 = least intense) with a different superscript letter in the same column of each taste modality are significantly different (*p* < 0.05) based on the RIX results in Table 3 and Table 4. Calculated based on 20 panelists × six independent replications.

**Table 6:** Intensity scores of saltiness and bitterness perceptions of the mixed salt solutions at different concentrations using PPM.

Taste	Sample ^†^	KCl:NaCl:L-Arg	Intensity Scores ^‡^		
			0.5% *w*/*v*	1.0% *w*/*v*	1.5% *w*/*v*
Saltiness	A	70:20:10	15.38 ^a, B^	36.23 ^b, A^	48.19 ^c, A^
	B	65:25:10	11.54 ^a, C^	38.60 ^b, B^	58.11 ^bc, A^
	C	60:30:10	12.60 ^a, C^	30.75 ^b, B^	66.61 ^ab, A^
	D	55:35:10	13.18 ^a, C^	38.07 ^b, B^	64.75 ^ab, A^
	E	0:100:0	23.60 ^a, C^	56.94 ^a, B^	79.27 ^a, A^
Bitterness	A	70:20:10	37.27 ^a^	41.61 ^a^	45.67 ^a, ns^
	B	65:25:10	26.32 ^a^	39.59 ^ab^	40.22 ^a, ns^
	C	60:30:10	33.19 ^a^	45.48 ^a^	39.03 ^a, ns^
	D	55:35:10	24.49 ^a^	30.27 ^ab^	40.36 ^a, ns^
	E	0:100:0	21.87 ^a^	23.13 ^b^	17.05 ^b, ns^

^†^ See Table 1 for detailed sample description. ^‡^ Based on thirty panelists and a 0 (no) to 100 (strong) scale. For each taste modality, intensity scores with a different lowercase letter in the same column indicate significant differences between samples based on Tukey’s HSD test (*p* < 0.05). For each taste modality, intensity scores with a different capitalized letter in the same row indicate significant differences between concentrations based on Tukey’s HSD test (*p* < 0.05). ns = not significant.

**Table 7:** The number of pairs with significant differences between the mixed salt solutions using RIX and PPM.

Salt Concentration ^†^	Saltiness Perception		Bitterness Perception	
	RIX	PPM	RIX	PPM
0.5% *w*/*v*	5	0	4	0
1.0% *w*/*v*	8	4	8	2
1.5% *w*/*v*	7	3	7	4
Total	20	7	19	6

^†^ Based on 10 different pair comparisons for each concentration. The number of pairs with significant differences for RIX and PPM were based on the results presented in Table 5 and Table 6, respectively.

## Data Availability

The original contributions presented in the study are included in the article. Further inquiries can be directed to the corresponding author.
